# Heparin-induced Thrombocytopenia: Pathophysiology, Diagnosis and Management

**DOI:** 10.7759/cureus.7385

**Published:** 2020-03-24

**Authors:** Vasileios Patriarcheas, Antonios Pikoulas, Minas Kostis, Andriani Charpidou, Evangelos Dimakakos

**Affiliations:** 1 Internal Medicine, Thoracic Diseases General Hospital Sotiria, Athens, GRC; 2 Internal Medicine, University Hospital of Heraklion, Heraklion, GRC; 3 Internal Medicine, University Hospital of Patras, Patras, GRC

**Keywords:** heparin-induced thrombocytopenia, hit, thrombosis, heparin, pf4, pf4/h complexes

## Abstract

Heparin-induced thrombocytopenia (HIT), even rare, is a life-threatening, immune-mediated complication of heparin exposure. It is considered the most severe non-bleeding adverse reaction of heparin treatment and one of the most important adverse drug reactions. The pathophysiological basis of HIT results from the formation of an immunocomplex consisting of an auto-antibody against platelet factor 4 (PF4) - heparin complex, which binds to the surface of platelets and monocytes, provoking their activation by cross-linking FcgIIA receptors. Platelets and monocyte activation, leads to the generation of catastrophic arterial and venous thrombosis, with a mortality rate of 20%, without early recognition. The definitive diagnosis of HIT i.e., clinical and laboratory evidence, can not be done at the onset of symptoms because laboratory results may not be available for several days. Thus, the initial approach is to predict the likelihood of HIT, because in highly suspected patients immediate heparin cessation and initiation of alternative anticoagulation treatment are crucial for the prevention of the devastating thrombotic sequelae. Herein, we describe the pathophysiology, the clinical manifestations, the diagnostic approach, and the management of patients with HIT.

## Introduction and background

It has been 100 years since the discovery of heparin by Dr. Henry Howell and 90 years since the introduction of heparin in clinical practice [1]. Up until today, it remains in widespread clinical use as a parenteral anticoagulant. The term heparin was introduced by Dr. Howell and is derived from the Greek root “hepar” i.e., the liver, the tissue where heparin was first produced. Heparin is a naturally occurring sulfated polysaccharide, with a molecular weight of 3.000 to 30.000 Da, whose main function is to inhibit blood coagulation [2].

Despite its characterization as an anticoagulant, heparin does not exhibit anticoagulant action per se. It binds through a pentasaccharide sequence to antithrombin a plasma serine protease inhibitor and enhances its antithrombotic activity to deactivate thrombin (factor IIa) and factor Xa. Heparin is administered only by the parenteral route, including both intravenously (IV) and the subcutaneously (SC) in order to treat or to prevent thromboembolic events, as well as for systemic anticoagulation during surgery [3].

Heparin therapy is associated with adverse effects, most commonly with the hemorrhagic complications ranging from life-threatening such as intracranial or retroperitoneal bleeding to hematomas at the injection site [4, 5]. Nonbleeding complications include osteoporosis in long-term treatment such as in women with high-risk pregnancies, delayed cutaneous hypersensitivity reactions, and heparin-induced thrombocytopenia (HIT) which is considered to be the most severe nonbleeding adverse reaction and one of the most important adverse drug reactions [6-8].

## Review

Heparin-induced thrombocytopenia

HIT has been categorized into two types: HIT type I and HIT type II.

HIT Type I

HIT type I, which is also known as heparin-associated thrombocytopenia (HAT), is a non-immune mediated response to heparin therapy. HIT type I is more frequent than type II, and it occurs in 10-30% of patients after heparin treatment [9]. Its typical presentation includes mild thrombocytopenia (rarely below 100.000/mm^3^) within the first two days of treatment. It is a self-limited direct effect of heparin and normalization of platelet count occurring spontaneously without discontinuation of therapy [10].

HIT Type II

Heparin-induced thrombocytopenia (HIT) type II is an immune-mediated adverse effect and represents a potentially catastrophic complication in which the administration of heparin has to be discontinued as soon as possible at the time of clinical suspicion [11].

It commonly develops after five to ten days of treatment and manifests with more severe thrombocytopenia (<100.000 /mm^3^) or a decrease in platelet count to less than 50% of baseline values [12]. HIT type II occurs with a frequency of 0.5% to 5% of patients treated with unfractionated heparin.

Risk factors for HIT type II can be categorized into drug- or host-related factors. Host-related risk factors include sex and age. According to Warkentin et al., there is a higher predisposition - twice the risk - for HIT development in females in comparison with males [13]. In another study, it was shown that HAT is rare among patients aged <40 years [14]. Drug-related risk factors include the type of heparin used (unfractionated heparin [UFH], low-molecular-weight heparin [LMWH]), and the duration of treatment. A study has shown that patients receiving UFH are five to ten times more likely to develop HIT compared to patients receiving LMWH, especially in therapeutic doses [12]. The duration of treatment also has to be considered a risk factor as it has been shown that shorter duration of exposure, is associated with a lower risk for HIT [15].

Pathophysiology

Platelet Factor-4 (PF4)

Despite the fact that the first reports for embolic events following heparin treatment were published in the late 1950s, the pathogenesis of HIT was only revealed in the early 1970s [16, 17]. 

The responsible antigen for the development of HIT is platelet factor 4 (PF4). Platelet factor-4 has various biological roles and is implicated in several cellular processes such as coagulation, hematopoiesis, inflammation, immune cell maturation [18-20]. PF4 is a 7.8kDa, 70-amino acid in length chemokine receptor (CXC), produced from megakaryocytes and released from platelet alpha-granules [21].

It is well known that PF4 neutralizes heparin action. This is achieved via the high-affinity binding on heparin due to the positive charge of PF4 and the negative charge of heparin. Thus heparin molecule has no more the ability to enhance the activity of antithrombin. 

However, the problem does not occur due to the heparin inactivation. Binding of PF4 to the heparin molecule opens the pandora box. Immunoglobulin G (IgG) antibodies are formed against the PF4/heparin complex leading to the creation of an immunocomplex which binds to the surface of platelets and monocyte and provokes their activation by cross-linking FcgIIA receptors [22].

Platelet activation leads to their aggregation as well as to the generation of microparticles with procoagulant activity, whereas monocytes activation promotes tissue factor (TF) expression, a glycoprotein that interacts with plasma factor VII/VIIa [23, 24]. TF/VII complex enables the initiation of the coagulation cascade via the proteolytic activation of factors IX and X, leading to thrombin formation [25].

Thrombocytopenia that occurs in HIT is moderately severe (bleeding is uncommon), and the main cause of clinical problems is the elevated levels of thrombin, which can give rise to life-threatening thrombotic sequelae like pulmonary embolism, mesenteric ischemia, ischemic limb necrosis, acute myocardial infarction, and stroke [9, 25].

Clinical presentation

The main clinical presentation of HIT is thrombocytopenia with or without thrombosis. Despite the fact that these two features are the keys for diagnosis, there are many other clinical situations which can result in low platelet count as well as thrombotic events such as HIT type I, massive pulmonary embolism, disseminated intravenous coagulation (DIC)/sepsis etc. [26,27]. Differential diagnoses can be done taking into account risk factors, time of the onset of thrombocytopenia, clinical symptoms, and laboratory findings.

Thrombocytopenia

Low platelet count is the cardinal manifestation of HIT. The majority of patients (~95%) exhibit thrombocytopenia with a decrease in platelet count more than 50% of the baseline or below 150.000/mm^3^ (mean nadir, 55.000/mm^3^) [28]. Thrombocytopenia can be the only manifestation of the disease (isolated HIT). Even though in these patients, there is a high risk (up to 50%) for subsequent thrombosis [29].

Thrombosis

Thrombosis is the most severe complication of HIT and can affect any vascular bed. According to a study by Warkentin, venous thrombosis occurs more frequently than arterial thrombosis; however, there are exceptions on this rule, and prompt investigation in patients with presumed HIT is crucial in order to prevent more serious and life-threatening complications [30]. Table 1 shows the most common sites of venous and arterial thrombotic complications [31].

**Table 1:** Thrombotic complications of heparin-induced thrombocytopenia Source: [31]

Venous	Arterial
Deep vein thrombosis	Aortic occlusion
Pulmonary embolism	Acute thrombotic stroke
Cerebral dural sinus thrombosis	Myocardial infarction
Adrenal hemorrhagic infarction	Thrombosis in upper limb, lower limb, mesenteric, renal and spinal arteries

Unusual Sequelae

Unusual clinical sequelae, including heparin-induced skin lesions and bilateral adrenal hemorrhage due to adrenal vein thrombosis, should be considered as manifestations of HIT syndrome [32, 33]. 

The Importance of the Onset of Symptoms

The onset of symptoms relative to heparin therapy is very crucial for the assessment of diagnosis [34]. As already mentioned, the pathogenesis of HIT involves the formation of IgG antibodies against the PF4/heparin complex leading to the creation of an immunocomplex which will activate platelets and monocytes. The presence of these anti-PF4/heparin antibodies is unusual in healthy individuals [35].

Warkentin and his colleagues found that the average time for antibody detection in heparin-naive patients is about four days from the start of therapy with thrombocytopenia and/or thrombosis occurring from day 5 to day 14 [36]. This is the typical onset. In patients who have had heparin treatment during the last 100 days, the onset is rapid, with a sharp decrease in platelet counts within 24 hours. On the other hand, HIT can occur days or weeks after heparin cessation. This situation is described as delayed onset HIT (D-HIT) [9].

Diagnosis

Thrombocytopenia is a rather common manifestation in patients treated with heparin. But as mentioned before, treatment with heparin does not always lead to HIT so there is an increased need for an accurate tool to distinguish patients at risk for developing the syndrome from patients who have thrombocytopenia due to other causes.

There are several scoring systems which can help physicians to predict the likelihood of HIT. The most widely used is the “4Ts” pretest scoring system (Table 2) [37]. The 4Ts scoring system is used to estimate the likelihood (pretest probability) of HIT, based on the available clinical features of patients receiving heparin treatment. The 4Ts scoring system is used to make a presumptive diagnosis until laboratory results are available. It was named after the four factors taken under consideration: **t**hrombocytopenia (percentage of platelet count drop), **t**ime/onset of thrombocytopenia (related to heparin exposure), **t**hrombosis (or other complications) and other possible causes of **t**hrombocytopenia. Each factor has a 0-2 scale with a maximum value of 4T score being 8.

**Table 2:** The 4Ts scoring system PLT - platelet Source: [37]

4Ts	2 points	1 point	0 point
Thrombocytopenia	Fall > 50% & PLT nadir > 20	Fall 30-50% or PLT nadir 10-19	PLT fall < 30%
Onset of PLT count fall	Onset between 5-10 days or PLT fall < 1 d	Consistent with days 5-10 fall but not clear; onset after 10^th^ d	PLT count fall < 4 d without recent exposure
Thrombosis or other sequelae	New thrombosis; skin necrosis	Progressive or recurrent thrombosis; skin lesions (not necrotic); not proven thrombosis	None
Other causes of thrombocytopenia	None apparent	Possible	Definite

Patients based on their score are categorized into three different probability sub-groups:

1. The low-risk group has a score of 0-3 with a low probability of developing antibody mediated-HIT (<5%).

2. The intermediate-risk group with a score of 4-5.

3. Patients having a 4T score of 6-8 are in the high-risk group. The risk of developing HIT in this group exceeds 80%.

While it is quite clear whether the low and high-risk groups are in danger of developing HIT or not, the intermediate-risk group is the most uncertain. For patients in this group, the laboratory detection of autoantibodies will be the most crucial factor that will determine the therapeutic approach that the physician will follow [13]. However, physicians have to take into consideration the possible delays in laboratory results.

The interesting point is that patients with no clinical evidence of thrombosis who fulfill other clinical characteristics of HIT can have a 4T score of 6, thus justifying the use of alternative anticoagulants [45].

The 4Ts scoring system has an increased negative predictive value of 99,8% for the low-risk group, while the intermediate and high-risk groups have a positive predictive value of 14% and 64%, respectively. Physicians have to take into consideration that incorrect or missing information of the 4T factors can lead to false scores and, consequently, bad decision making and incorrect therapeutic approach in patients with HIT. Although informative and useful in clinical practice, the 4Ts scoring system has limitations in the intensive care unit (ICU) patients and in the management of patients undergoing cardiac surgery [10].

In patients who are categorized as having intermediate or high-risk for HIT based on their score (>=4), heparin should be discontinued, and alternative anticoagulant medication has to be started. HIT antibodies have a high affinity for LMWH, and for this reason, LMWH has to be excluded from the available therapeutic options for these patients [10].

Laboratory Investigations

Initial management has to be according to the probability of HIT in order to prevent major complications and avoid the increased risk of mortality, taking into account that laboratory results may not be available. Laboratory investigation has a complementary role to diagnosis and should be performed only in patients with clinical suspicion of HIT, i.e., patients with intermediate and high probability scores [38].

There are immunoassays and functional assays for the detection of HIT antibodies. Immunoassays are performed using an enzyme-linked immunosorbent assay (ELISA) technique. The patient’s serum is added into microwells coated with PF4/polyvinyl sulfonate complex. In the presence of antibodies against this complex, binding will occur. A second labeled antibody is used in order to confirm binding and, by extension, the presence of HIT antibody. Immunoassays, on the one hand, have the advantage of sensitivity (>99%), but on the other hand, lack specificity (40-70%), and for this reason, several different technical approaches have been developed for their improvement [39, 40].

Functional assays include the serotonin release assay (SRA) and the heparin-induced platelet activation (HIPA). In these assays, donor platelets are incubated with heparin and patient plasma in order to measure HIT antibody-induced platelet activation. Functional assays have high specificity (>95%), but exhibit a wide range in sensitivity from 60% to 100% [40, 41].

Management

The two pillars of HIT management are immediate heparin cessation and initiation of alternative anticoagulation treatment. Patients who are categorized as intermediate or high-risk based on their 4T score (>=4) are discontinued from heparin. Heparin administration should be avoided from any source; for this reason, heparin-coated catheters should be removed. Cessation of unfractionated heparin is not sufficient to prevent thrombotic events, and an alternative anticoagulant medication should be provided, with the exception of both LMWH or warfarin, which can induce thrombin generation and increase the risk for thrombosis [42, 43]. Alternative anticoagulation therapy involves direct thrombin inhibitors such as argatroban, bivalirudin, or factor Xa inhibitors such as danaparoid and fondaparinux.

Direct Thrombin Inhibitors

Argatroban is a synthetic direct thrombin inhibitor that reversibly binds to both free and clot-bound thrombin. It is approved by the Food and Drug Administration (FDA) in the United States for the treatment of HIT, as well as for anticoagulation in patients undergoing percutaneous coronary intervention (PCI) when heparin is contraindicated [44, 45]. Its administration is via continuous IV infusion at a dose of 2 μg/kg/min, and a steady-state is achieved within one to three hours. Treatment with argatroban is monitored by measuring, activated partial thromboplastin time (target range 1.5-3 the baseline activated partial thromboplastin time [aPTT]) [46].

Bivalirudin is also a synthetic-hirudin analog and direct thrombin inhibitor that binds to both free and clot-bound thrombin. It is used during cardiac surgery in patients with an active or recent history of HIT and as a possible alternative for the treatment of HIT [47]. In contrast to argatroban, which exhibits hepatobiliary clearance, bivalirudin clearance is achieved renally or by enzymatic degradation. The administration is via continuous IV infusion at a dose of 0.15mg/kg/h.

Factor Xa Inhibitors

Danaparoid is non-heparin, low- molecular-weight, sulfated glycosaminoglycans that exhibit long-acting antithrombin-dependent anti-Xa activity. It has been approved for the treatment of HIT (none in the United States) and does not cross the placenta. Danaparoid is given intravenously with an initial bolus dose according to the patient’s weight [9]. Treatment with danaparoid is monitored by measuring anti-Xa activity.

Fondaparinux is a synthetic analog of the antithrombin-binding pentasaccharide sequence, a synthetic antithrombin-dependent factor Xa inhibitor [48]. Despite the fact that fondaparinux has not been approved for HIT treatment, it has several advantages over direct thrombin inhibitors such as once-daily subcutaneous administration and no requirement for routine anti-Xa monitoring [49].

Transition to Vitamin K antagonist

Heparin-induced thrombocytopenia is a recognized risk factor for subsequent venous thromboembolism; thus, anticoagulation treatment has to be continued for six weeks in patients with isolated HIT and for three months in patients with thrombotic sequelae. Early administration of warfarin should be avoided due to the risk for warfarin-induced skin necrosis and venous gangrene and should be slowly introduced only when patient’s platelets have returned to normal levels [45].

Shifting from a direct thrombin inhibitor - especially argatroban - to a vitamin K antagonist (VKA) remains a challenge for the clinician. There should be an overlapping period for five days between VKA and the chosen parenteral anticoagulant, and it is well known that argatroban leads to a profound increase of international normalized ratio (INR), making the transition difficult [50].

## Conclusions

Heparin-induced thrombocytopenia remains a rare complication with high morbidity and mortality in patients receiving heparin treatment. The cardinal symptom of HIT is thrombocytopenia, a feature that is common among hospitalized patients under heparin treatment, thus it requires a high index of suspicion in order to confirm or to exclude HIT development. When HIT is suspected, heparin cessation and treatment with direct thrombin inhibitors or factor Xa inhibitors, are sine qua non for the prevention and management of thrombotic events.
